# DNA Amplification: Current Technologies and Applications

**DOI:** 10.3201/eid1102.041049

**Published:** 2005-02

**Authors:** Robert F. Massung

**Affiliations:** * Centers for Disease Control and Prevention, Atlanta, Georgia, USA

**Keywords:** DNA Amplification, book review, polymerase chain reaction, PCR

DNA amplification is a powerful technique that has had an immense impact on scientific research in the past 2 decades. While polymerase chain reaction (PCR) is still the most popular method, alternative methods of DNA amplification are constantly being developed. In addition, the extraordinary versatility of PCR has led to its use in novel ways that have opened new avenues of research. These novel methods for DNA amplification and the versatility of PCR are highlighted in DNA Amplification: Current Technologies and Applications.

The 17 chapters in this book are divided into 4 sections that focus on enzymes (3 chapters), thermal cycling methods (6 chapters), isothermal methods (6 chapters), and the detection of non-DNA analytes by DNA amplification (2 chapters). Each chapter has a thorough description of methods and highly detailed protocols for applying the technique to at least 1 specific application. Several excellent chapters describe the uses of Phi29 DNA polymerase and of applications using isothermal rolling circle amplification. A chapter on multiple-displacement amplification details the isothermal amplification of total genomic DNA and should prove extremely useful for amplifying DNA in limited amounts, such as DNA from clinical samples. The final 2 chapters describe use of either real-time PCR or rolling circle amplification to detect and quantify non-DNA analytes, such as serum cytokines, with much greater sensitivity than conventional enzyme-linked immunosorbent assay methods.

This book is not for the novice scientist, as it does not describe basic DNA amplification fundamentals; rather, it is directed at those with a solid background in molecular biology who desire knowledge of cutting-edge applications. Although many of the detailed protocols will not be applicable to certain laboratory situations, the versatility of most of the methods described will allow them to be easily adapted to other studies. Therefore, this book will be a good addition to the library of researchers in molecular biology or to molecular diagnostics laboratories planning to expand their horizon beyond standard PCR amplification techniques.

